# Supplementary material for: Testing of a novel questionnaire of Household Exposure to Wood Smoke
Source: PLOS Glob Public Health. 2023 Jan 25;3(1):e0001500. doi: 10.1371/journal.pgph.0001500 (PMC10022189; doi:10.1371/journal.pgph.0001500)
Supplement: Attachment — Submitted filename: Response to Reviewers_table_12.6.22.docx [file pgph.0001500.s001.docx]

**Response to journal comment**

| **Reviewer 1 Comments** |  |
| --- | --- |
| My main comment is about the possibility that measurement error may not be adequately captured by the factor analysis. Namely, I am curious about whether adding a methods factor to the measurement model in a CFA would reveal different associations between items and perhaps even support a two-factor solution where the methods factor is accompanied by a substantive factor (the HEWS scale). The sample size is definitely on the smaller end for CFA but I would like for the authors to explore the possibility that the different response options (“Never to Always”vs. the duration response options vs. the distance response option) are a possible source of measurement error that can be picked up and parallel out. | Thank you for this comment. We agree that it would be interesting, but we have chosen not to conduct this analysis at this time for two reasons; 1) the item to item correlation matrix does not support a relationship associated with the scale wording (see text pg X and supplemental information), 2) we believe that the sample size is not sufficient to adequately conduct the CFA to detect measurement error as a result of the response options. In addition to noting the potential for methods variance and interpreting the item level correlations in light of this (results p. 13), we acknowledge the importance of future research, including CFA, to further examine this possibility (discussion p.21). |
| It would be interesting to add a table with the raw correlations between each individual item in addition to the correlation between each item and the rest of the items. This additional table could perhaps go in the appendix. | We agree and have included the correlation matrix as supplemental information. |
| Why is it not possible to make the replication data available to the journal? They could be  appropriately deidentified so as to protect human subjects and comply with the standards of the journal and the the academic community at large. | We have deidentified all data used in this article and uploaded the material to an appropriate database. |
